# Lessons learned from the prenatal microbiome controversy

**DOI:** 10.1186/s40168-020-00946-2

**Published:** 2021-01-12

**Authors:** Martin J. Blaser, Suzanne Devkota, Kathy D. McCoy, David A. Relman, Moran Yassour, Vincent B. Young

**Affiliations:** 1 Departments of Medicine and Pathology and Center for Advanced Biotechnology and Medicine, Rutgers University, New Brunswick, USA; 2 F. Widjaja Foundation Inflammatory Bowel and Immunobiology Research Institute, Cedars-Sinai Medical Center, Los Angeles, California USA; 3 Department of Medicine, David Geffen School of Medicine, University of California-Los Angeles, Los Angeles, California USA; 4 Department of Physiology & Pharmacology, Cumming School of Medicine, University of Calgary, Calgary, Alberta Canada; 5 Departments of Medicine, and of Microbiology & Immunology, Stanford University, Stanford, USA; 6 Section of Infectious Diseases, Veterans Affairs Palo Alto Health Care System, Palo Alto, USA; 7 Chan Zuckerberg Biohub Microbiome Initiative, San Francisco, California USA; 8 Microbiology and Molecular Genetics Department, Faculty of Medicine, The Rachel and Selim Benin School of Computer Science and Engineering, The Hebrew University of Jerusalem, Jerusalem, Israel; 9 Department of Internal Medicine/Division of Infectious Diseases, Department of Microbiology & Immunology, The University of Michigan, Ann Arbor, USA

## Abstract

For more than a century, the prenatal environment was considered sterile. Over the last few years, findings obtained with next-generation sequencing approaches from samples of the placenta, the amniotic fluid, meconium, and even fetal tissues have challenged the dogma of a sterile womb, and additional reports have emerged that used culture, microscopy, and quantitative PCR to support the presence of a low-biomass microbial community at prenatal sites. Given the substantial implications of prenatal exposure to microbes for the development and health of the host, the findings have gathered substantial interest from academics, high impact journals, the public press, and funding agencies. However, an increasing number of studies have challenged the prenatal microbiome identifying contamination as a major issue, and scientists that remained skeptical have pointed to inconsistencies with in utero colonization, the impact of c-sections on early microbiome assembly, and the ability to generate germ-free mammals. A lively academic controversy has emerged on the existence of the wider importance of prenatal microbial communities. *Microbiome* has asked experts to discuss these issues and provide their thoughts on the implications. To allow for a broader perspective of this discussion, we have specifically selected scientists, who have a long-standing expertise in microbiome sciences but who have not directly been involved in the debate so far.

**About the authors:**

**Martin Blaser** is Director of the Center for Advanced Biotechnology and Medicine and Henry Rutgers Professor of the Human Microbiome at Rutgers University. He is a specialist in Infectious Diseases, and studies the effects of perturbations of the microbiota on the eventual development of disease to identify ways to restore heath.

**Suzanne Devkota** is the Director of Microbiome Research at the Cedars-Sinai Medical Center and Assistant Professor in the Department of Medicine at the University of California, Los Angeles. Her primary research areas include the study of dietary influences on gut microbiome structure and function, and she investigates gut microbiome drivers of Crohn’s disease complications.

**Kathy McCoy** is a Professor in the Cumming School of Medicine and Scientific Director of the International Microbiome Centre. She is a mucosal immunologist that studies how the microbiome impacts the development of the immune system and regulates immune responses. Her lab utilizes gnotobiotics to interrogate host-microbiome interactions and to define the cellular and molecular mechanisms by which the microbiome shapes immunity.

**David Relman** is the Thomas C. and Joan M. Merigan Professor in the Departments of Medicine, and of Microbiology & Immunology at Stanford University, and Chief of Infectious Diseases at the Veterans Affairs Palo Alto Health Care System in Palo Alto, California. He was an early pioneer in the use of modern molecular methods to study the human microbiome. His current research seeks to identify processes that underlie community assembly, stability and resilience in the human microbial ecosystem and contribute to human health and disease.

**Moran Yassour** is an assistant professor at the Hebrew University Faculty of Medicine and Computer Science and Engineering School. The Yassour lab studies the establishment and development of the infant gut microbiome, how delivery mode and feeding practices impact the infant microbial dynamics and composition. The lab specializes in generating birth cohorts, and uses computational and experimental approaches to infer mother-to-child microbial transmissions.

**Vincent Young** is the William Henry Fitzbutler Collegiate Professor at the University of Michigan School of Medicine. As an infectious diseases physician/scientist he has a long-standing interest in gastrointestinal bacterial infections. He has a focus on hospital acquired infections, including *C. difficile* and their relationship with the host and the indigenous microbiota.

Q: *Do you think the available scientific evidence is in favor of the ‘in utero colonization’, or the ‘sterile womb’ hypotheses? Do you see a need for additional experimental work to address this controversy?*

**Martin J. Blaser.** There is no part of the human body that is universally sterile at all times. Adventitious infections can occur anywhere. For example, during pregnancy, an acute or chronic infection of the mother can cross the placenta, directly infecting the fetus, as in the case of congenital rubella (viral), syphilis (bacterial), or toxoplasmosis (protozoal). However, these are exceptional events. Just like the brain, the placenta has important barriers to the spread of microbes, so that the critical processes will not be disturbed. That the placenta is essentially sterile in mammals is supported by the studies of xenobiosis—the ‘germ-free state’. The development of a germ-free line depends on the founding members being born by Cesarean-section, and continued in xenobiosis to breed. Based on all conventional ascertainment methods such animals, and the line of their progeny, are sterile. If there was a microbiota, it likely would be propagated from generation to generation. Xenobiosis has been achieved in rodents, ungulates, swine, and humans, among other species, which counters the notion that there is an indigenous placental microbiota. The studies of human babies born by C-section have shown varying results, which likely can be accounted for by differences in methodology and interpretation. Any claim that there is indeed an indigenous microbiota would need to be well-substantiated and unequivocal, since it would need to surmount both existing theory and logic. At this point, no finding has passed that threshold, in my opinion.

**Suzanne Devkota.** Colonization vs identifying a true microbial niche are two different questions. Colonization can be transient or persistent, but a true microbial niche implies a sophisticated, evolved, host-microbe state. The available work so far suggests it is certainly possible that microbes are in the in utero environment, but I believe at best these microbes come from somewhere else in the body and are not native to the in utero environment. It’s hard for me to reconcile these human studies proposing a placental microbiome with the fact that germ-free animals exist. On the other hand, you have recent work by Susan Lynch’s group showing colonization of the fetal gut, which was compelling, and a very difficult study to run. There is always something to be learned from all of these studies- even if only to shine a light on the fact that reagents can be contaminated, as has been put forth to counter some earlier placental microbiome studies. Ultimately, whenever exploring a microbial presence in sites we believe to be “sterile”, the burden of proof is very high, as it should be. More work needs to be done.

**Kathy D. McCoy.** I believe that the currently available scientific evidence is more in favor of the ‘sterile womb’ hypothesis. The majority of evidence thus far does not support the presence of a bona fide resident microbial population in utero. However, it is possible that there are incidences of transient microbial exposure in utero, especially in humans that have a long gestational period. Additional experimental work is required to address this controversy. It is important to understand whether there is limited or transient microbial exposure in utero*,* the extent to which microbial products or metabolites pass the placenta, and whether this plays an instructive role in immune development.

**David A. Relman.** Before answering the questions, clarification of terms and language may be in order. ‘Colonization’ typically refers to establishment of a plant, animal, or microbe in a habitat, usually in a persistent manner for some period of time, typically reinforced by interactions with pre-existing residents, a host, or with the abiotic environment. ‘Sterile’ simply means free of life, but sterility is traditionally defined by the failure of cultivation methods to reveal evidence of life. These points are important, because they focus the discussion.

With these comments in mind, currently available scientific evidence does not support true in utero microbial colonization by a consistent species or set of species during normal states of health. Furthermore, there is no support for the concept of a true “microbiota” in the placenta or in the amniotic sac. ‘Microbiota’ refers to a microbial community, and a community from an ecological perspective is a set of interacting and often interdependent species.

What kinds of evidence prompted this controversy? For the most part, it was the detection of bacterial DNA in placental tissue, including the fetal membranes, by PCR. But the presence of bacterial DNA has been highly inconsistent across studies and investigators. The studies with the most rigorous controls and most robust design have found no microbial DNA, other than the sequences of an occasional, known pathogen. A few studies report detection of bacterial DNA in placental tissue with fluorescent in situ hybridization. However, these data as well are sparse, inconsistent, and unconvincing. A reasonable alternative explanation for the detection of low levels of bacterial DNA in these clinical samples is contamination--of PCRs, tissues, or reagents. Another possible explanation is the true presence of bacterial DNA in the blood of subjects, and the amplification of this DNA from these blood-rich tissues. Bacterial translocation or leakage of bacterial components including DNA into so-called ‘privileged’ anatomic sites of the human body, such as the amniotic sac and placenta, may occur in some individuals, even in health, but is likely rare. We showed a decade ago that bacterial DNA can be detected in the amniotic fluid of a small subset of pregnant women and that it may be a predictor of future adverse gestational outcomes: its abundance correlated with IL-6 levels and leukocyte counts in amniotic fluid, and inversely with time to delivery. Most importantly, the presence of DNA is quite distinct from ‘bacterial colonization’ and very different from the presence of a true ‘microbiota’. Both contamination and the presence of bacterial DNA in blood are plausible explanations for the controversial findings at hand. Additional carefully controlled studies that provide further clarity about the origins and nature of these two phenomena would be helpful.

**Moran Yassour.** As I see it today, most published evidence supports the ‘sterile womb’ hypothesis. While some studies suggest in-utero colonization, their evidence does not indicate a live and growing community but rather suggests presence of DNA fragments. The challenge, of course, lies in the extreme low biomass of potential in utero colonization, and thus the signal to noise can be very challenging. To rigorously evaluate the microbial presence at such body sites, one must have clear negative controls added at each step of the process, especially accounting for various contaminants from reagents, etc. The extra mile is to also include positive controls to evaluate quantity as well, as was done by de Goffau et al. [1], where spike-ins were added.

**Vincent B. Young.** I think that before we even start addressing this controversy it would be good to define what I would consider to be a placental microbiome. I know that there are multiple definitions of microbiome but for my answers I will refer to the microbiome as “A characteristic microbial community that occupies a reasonably well-defined habitat and has distinct physicochemical properties.” When referring to the microbes that form a community itself, I will use the term microbiota. The reason for using these definitions is that I think that in order to address the controversy I think that the bar is pretty high for demonstrating the presence of a placental microbiome. For me, simply demonstrating that you can detect microbes (and mostly this has focused on bacteria, so I will restrict my considerations for this, knowing that we should also address potential viral or fungal microbiota) by culture-independent methods (e.g. 16S rRNA libraries or shotgun metagenomic sequencing), fluorescent in situ hybridization (FISH) or even bacterial culture isn’t enough. In my opinion, you need to show that this potential community is stable over time, reproducing in situ and is metabolically active. As of yet, I haven’t seen evidence that supports this. People may argue that I am setting an unreasonable expectation, but this is what I favor.

Despite these considerations, even if the womb is sterile (meaning that there is not a viable, reproducing, metabolically active microbial community present) I think it is likely there are microbial influences on the developing fetus. The interface here is again the placenta. The presence of microbes or microbial products that investigators have detected in the placenta, even if not viable, can serve to educate the fetus through immunologic or chemical (i.e. small molecules) exposure to the mother’s indigenous microbiota. It is possible that this exposure to microbial products can play a critical role in the development of the fetus. I realize that some have used our ability to generate germ free mice to argue against the ‘sterile womb’ hypothesis. It is hard to argue against this. However, it is interesting to consider if the differences in the immune system seen in germ free animals has not so much to do with the lack of microbiota in these animals per se but reflects the abnormal development that results from having a germ-free mother. The reason to make these arguments is to return to the point that the argument as to whether the womb is sterile may cloud the important questions as to the role microbial exposure (either directly or indirectly) in the health and development of the fetus.

Q: *Did studies of the prenatal microbiome provide novel perspectives for the field at large? Do you see lessons that should be drawn from prenatal microbiome research and how it will affect the study of other body sites considered sterile?*

**M.J.B.** To prove sterility is a more difficult task than to recognize the presence of a microbe. Sterility requires the absence of any agent, but there likely are infectious/microbial agents that have not yet been discovered. Thus, as predicted by the “Uncertainty Principle” in physics, proving sterility is an elusive goal. Nevertheless, it is possible to rule-in or rule-out the presence of broad classes of microbes when standard/conventional methodologies are used. In fact, the agents that have been claimed to be present in the parturient human placenta are ones that are easy to verify or not, using standard tools. The absence of uniformity in the results in the literature suggests important differences and gaps in technical issues. Such issues have arisen and will arise again when researchers claim that a particular site that has believed to be sterile is not so. We need an equivalent to Koch’s postulates to set standard rules for indicating the presence of microbes in a space previously considered as ‘sterile’.

**S.D.** Yes, I think it raises the important question of what are true microbial niches in the body and what are the result of translocation and what is artifact (sample handing or reagent contamination)? I think in time we are going to learn that many parts of the body are far less sterile than we thought. However, truly inhabiting a human body site necessitates mutualistic interactions between the microbes themselves in a way that does not cause detriment to the host in steady state. It is important to distinguish this from translocation, which I do believe is quite common and can have an effect on host physiology (in ways we are still uncovering). But simply because these microbes travel does not beget a new niche wherever they happen to land. Some of the controversy can be attributed to disagreements about terminology. It’s important to shine a light on that. We also should always ask ourselves the simple teleological question “does it make sense?” given what we know about co-evolution and host physiology. For example, if microbes are purported to be colonizing a previously “sterile” tissue, does it make sense given what we know about immune responses in that tissue? Asking this does not preclude an openness to novelty, but is important in guiding the next set of experiments. You can’t just stop at showing presence or absence of microbial DNA.

**K.D.M.** I firmly believe that studies that challenge the existing dogma are beneficial to research; this is how science progresses. The studies of the prenatal microbiome certainly challenged microbiome researchers to optimize and control protocols for microbial detection. This controversy also led to important discussions of what is meant by the term ‘colonization’ and should stimulate microbiome researchers to better define the terms that are being used in their field. Should the presence of only a small number of bacteria, even if live, be considered a resident colonization? What are the functional consequences of the presence of microbial DNA, small numbers of live bacteria, or transient exposures during gestation? The controversy of a microbial presence at a site long considered to be sterile increases debate and discussion in the field, which ultimately will move microbiome research forward.

**D.A.R.** This controversy about a ‘placental microbiome’ highlights several important issues. First, as I mentioned earlier, microbial translocation into the peripheral bloodstream (and portal circulation) is generally believed to be a regular feature of health, and associated with local disturbances of skin and mucosa. Because translocation tends to be transient and rare, it is not surprising that consistent findings and patterns of microbial DNA in the blood of healthy people are difficult to establish. While microbial translocation may be important for the health of the host, it certainly complicates the study of ‘privileged’ anatomic sites. Again, we have to remember that DNA does not necessarily mean live or even intact organisms. Second, research findings increasingly support the idea that systemic trafficking of bacterial products, especially bacterial metabolites, during pregnancy and at the time of delivery may have impacts on the postnatal development of the infant. We need to become much better informed in general about the systemic distribution and vertical transmission of byproducts from maternal skin and mucosal microbial communities, and their roles in promoting the health of the child.

**M.Y.** At this point, the prenatal controversy definitely affects the field, and so does the breast milk controversy, as in both cases, trying to prove a low-biomass commensal colonization is very hard. This challenge of profiling low-biomass microbial communities is indeed an opportunity to lay out some groundbreaking research, on a very specific and debated topic, far from the next random-association shout-out. If we can effectively handle these low biomass/ low diversity microbiome environments with improved lab techniques and analyses, we would also handle complex microbial communities better.

Another lesson that is emerging is the sensitivity of 16S vs. metagenomic sequencing. Especially when handling samples from body sites that include many human cells, 16S has better sensitivity than metagenomics and should not be automatically disregarded as the less-advanced method.

**V.B.Y.** One observation on the studies of the prenatal microbiome is that from the outside, it appears that the results of microbiome research are very dependent on technical considerations. Having been involved in this line of research for almost 20 years, I have found it disturbing that other scientists feel that investigators studying host-associated microbes are constantly involved in “fishing expeditions” using increasingly expensive and technologically dependent methods. The studies of prenatal microbiota have come to represent, in certain scientific circles, the worst of the critiques from skeptical scientists.

To return to my previous points regarding the definitions of microbiome, when considering other body sites considered to be sterile (e.g. brain/central nervous system and blood) there are many studies that refer to the “blood microbiome” and the “brain microbiome.” Again, we need to consider what was observed and how these observations were made. Finally, we need to return to the potential biological effects of microbial exposure (be it if from viable organisms that are eventually cleared or from microbial products) on the host. There have been many position papers that have observed that studies of the microbiome have been moving from studies of *structure* (studies of ‘who is there’) to studies of *function* (studies of what the microbes are doing in terms of metabolism, immune interactions etc). I think that this type of research is important in studies of the prenatal microbiome. It is likely that the fetus is not completely “blind” to microbes. However, the exact manner in which the fetus is made aware of the microbial world and the results of this exposure can be the next focus of research. While the nature of exposure is still of interest, the research should not stop at that point. In my opinion, perhaps some of the heated debate over the nature of neonatal microbiome has delayed moving to these other important biologic questions.

Q: *How does the controversy on the prenatal microbiome affect the credibility of the microbiome research field?*

**M.J.B.** Acceptance of scientific findings is based on the trust of the scientific community and the general public in the honesty and accuracy of the scientists who report particular findings. Whenever that is not achieved, through dishonesty or the reporting of findings that later are shown to be incorrect, then the credibility of the scientific process is diminished. Thus, if work is reported with the emphasis of the novelty of the findings and especially its potential impact on human health, and later shown to be incorrect, the impact can be great. This is especially magnified by the many fringe groups that have their own scientific agendas, and who latch on to controversy as a way of disparaging the scientific edifice. Reasonable scientists can differ in their interpretations of the data, but the rock underpinning the scientific edifice is the correctness (and rigor) of the scientific observations themselves.

**S.D.** I don’t think it affects credibility of the field per se. Every field has controversy, that’s science.

**K.D.M.** I don’t believe that in the long-term this controversy will be detrimental to the credibility of the microbiome research field. However, credibility is linked to responsible reporting in general. The microbiome research field needs to continue to work together to search for the truth.

**D.A.R.** This controversy has certainly highlighted the understandable but unfortunate tendency of many investigators and the general public to embrace and accept too quickly, early and unconfirmed findings that are provocative or contrary to traditional teaching. The credibility of the microbiome field suffers, like other fields, when new findings are announced breathlessly and in haste, and a receptive but uncritical public (and sometimes scientific community) fails to question the findings and demand replication. The microbiome field, because of the scope and scale of the ongoing research effort, the rapidity of advances, and the interest of the public, may be especially vulnerable to this problem.

**M.Y.** Because of this controversy, the whole microbiome research field is talking more about contamination biases. This is a critical discussion, and we should all remind ourselves to (a) add more controls; and (b) search for the common contaminants in our results. Because the microbiome field is so hype, and there is not enough standardization, oftentimes there will be several published studies with contradictory results. The long-term consequence of such studies is that scientists consider the microbiome field as not rigorous enough, when for each study we can find another one that shows the opposite. This point was even covered in a piece in The Atlantic [Ed Young, April 2016]: “Thousands of studies have linked the microbiome to almost every condition you can imagine, but many of these correlations are likely to be illusory”. The strenuous open debate is a common “feature” of a new field, but the practices and standards have to converge at some point. This specific niche of the field (maternal-fetal microbiome) is also inherently sensitive to the biological/ethical implications of additional sampling, so if over-sampling cannot be a solution, there is space for some conceptual and technological advancement.

**V.B.Y.** As alluded to above, the microbiome research field in general may have been adversely affected by those who observe the debate from outside of the field. A colleague of mine once noted that this debate reminds him of the wondering “how many angels can dance on the head of a pin.” We need to make it clear that microbiome research is the continuation of the long and fruitful field of microbiology, which in this context is focused on host-microbe interactions. The debate over the prenatal microbiome is not simply a philosophical dispute but is part of a scientific exploration of potential microbial influences on the development and health of a developing fetus.

Q: *What are the responsibilities of the community of microbiome researchers, scientific journals, lay press, and funding bodies in safeguarding the credibility of the microbiome field?*

**M.J.B.** The responsibilities of all are the same: to conduct and report scientific research that is based on careful methodologies with proper controls in which the conclusions are properly based on the results of the study. In our current age, perhaps in all ages, there is a tendency to overstate the significance of scientific findings, because of the competition for attention and money. This is a bias and ultimately hurts our field by inaccurately assigning priorities, and directing resources in ways that are not optimally productive. This particular lesion is present in all fields of medical research, and perhaps reflects the Darwinian competition for resources. However, methodologies can be adapted that should minimize its impact.

**S.D.** The primary responsibility of the media and scientists alike, in my view, is to not overstate the impact of the microbiome. Don’t give people false hope, don’t fear-monger, and don’t claim the microbiome can tell us things that it can’t. Not prematurely at least. It’s an incredibly promising field, and there is a real potential for microbially-directed therapies, I believe that wholeheartedly, but there are still aspects of fundamental host-microbe biology we seem to have completely skipped over in order to do sexy science. These don’t have to be mutually exclusive. The power of multi-‘omics can’t be argued with, but the value of big data will always be stronger if tied to real physiology. In my view, that’s the best route to securing credibility of the field.

**K.D.M.** All parties must be accountable for responsible reporting in order to safeguard the credibility of the microbiome research field. Microbiome researchers must take care not to over-interpret their findings. Microbiome researchers also function as peer-reviewers and in this role, they must critically assess the use of the controls and methodology required for robust microbiome research. Scientific journals and editors must ensure vigorous and fair review of manuscripts and not be tempted to publish ‘sensational’ reports against reviewer’s recommendations. Scientific journals must also publish follow-up manuscripts that will help to expand the findings. The lay press must be very responsible in reporting findings to the public and must resist the temptation to sensationalize – and microbiome researchers must do their best in interviews to not allow this type of reporting or hype.

**D.A.R.** I view the responsibilities of the scientific community as paramount. We need to think more about the health of the general scientific enterprise and less about our own popularity ratings, number of ‘followers’ and ‘likes’. We need to be critical yet respectful, work tirelessly towards the goal of reproducible research, and cooperate fully in sharing protocols, workflows, and raw data. The value of negative findings from well-designed, carefully controlled studies, and of replication datasets must be promoted. The same responsibilities apply to journals, press, and funders, but in my view, it all starts with the scientist and the science.

**M.Y.** Our responsibility as a research community and publishing groups is (as always) to be critical of what we are presented with, and to carefully consider all study controls. The credibility of a field is safeguarded if clear guidelines are given for the standardization of practices. If a study comparing all variables related to the process (from sample collection, extraction, sequencing and analysis) was conducted, we could better evaluate the differences across papers and determine which are the best/worst practices in this regard. Such a study could be a good benchmark for all contributors to the field, that could evaluate research pipelines and compare their results with the ones from other papers. Pioneering studies of standardizing pipelines are starting to emerge, like Amos et al. [2], and it would be beneficial if additional such studies were conducted. Such an effort could be embarked by international consortia which already provide considerably large datasets to the field and have the credibility of setting a gold standard for the entire community.

Finally, a personal take home message for all microbiome researchers, when we establish a new cohort, we must include all the adequate controls, to go above and beyond to prove this is signal rather than noise.

**V.B.Y.** In sum, science thrives on debate, but debate should be the means to the end of advancing knowledge and not the end in and of itself. In my opinion, the important knowledge to be gained is understanding how the close symbiosis between a host and their indigenous microbes affects both parties involved. This understanding could lead to novel therapies and interventions to improve host health by fostering a mutually beneficial interaction between host and resident microbes.
